# Oxygen uptake, heart rate, perceived exertion, and integrated electromyogram of the lower and upper extremities during level and Nordic walking on a treadmill

**DOI:** 10.1186/1880-6805-32-2

**Published:** 2013-02-13

**Authors:** Koji Sugiyama, Mami Kawamura, Hisato Tomita, Shizuo Katamoto

**Affiliations:** 1 Deptartment of Health and Physical Education, Faculty of Education, Shizuoka University, 836 Ohya, Suruga, Shizuoka 422-8529, Japan; 2 Aichi Prefectural Saori Special Needs Education School, 37 Nakahigashiyama, Nishikawabata, Aisai City, Aichi, 496-8019, Japan; 3 Shizuoka Institute of Science and Technology, 2200-2 Toyosawa, Fukuroi, Shizuoka, 437-8555, Japan; 4 Graduate School of Health and Sports Science, Juntendo University, 1-1 Hiraga-gakuendai, Inzai City, Chiba, 270-1695, Japan

**Keywords:** Nordic walking, Oxygen uptake, iEMG, Lower extremity, Upper extremity

## Abstract

The purpose of this study was to characterize responses in oxygen uptake ( $\overset{\cdot }{V}O_{2}$), heart rate (HR), perceived exertion (OMNI scale) and integrated electromyogram (iEMG) readings during incremental Nordic walking (NW) and level walking (LW) on a treadmill. Ten healthy adults (four men, six women), who regularly engaged in physical activity in their daily lives, were enrolled in the study. All subjects were familiar with NW. Each subject began walking at 60 m/min for 3 minutes, with incremental increases of 10 m/min every 2 minutes up to 120 m/min $\overset{\cdot }{V}O_{2}$ , ${\overset{\cdot }{V}}^{E}$ and HR were measured every 30 seconds, and the OMNI scale was used during the final 15 seconds of each exercise. EMG readings were recorded from the triceps brachii, vastus lateralis, biceps femoris, gastrocnemius, and tibialis anterior muscles. $\overset{\cdot }{V}O_{2}$ was significantly higher during NW than during LW, with the exception of the speed of 70 m/min (*P* < 0.01). ${\overset{\cdot }{V}}^{E}$ and HR were higher during NW than LW at all walking speeds (*P* < 0.05 to 0.001). OMNI scale of the upper extremities was significantly higher during NW than during LW at all speeds (*P* < 0.05). Furthermore, the iEMG reading for the VL was lower during NW than during LW at all walking speeds, while the iEMG reading for the BF and GA muscles were significantly lower during NW than LW at some speeds. These data suggest that the use of poles in NW attenuates muscle activity in the lower extremities during the stance and push-off phases, and decreases that of the lower extremities and increase energy expenditure of the upper body and respiratory system at certain walking speeds.

## Background

Over the past decade, Nordic walking (NW) has gained in popularity as a healthy sports activity by northern Europeans. A recent study found that 10 million people participate in this sporting activity in various countries [1]. At a given walking speed, NW involves more muscles in various body segments and induces greater exercise intensity compared with level walking (LW) [2-11]. Furthermore, NW places a smaller load on the legs during walking [12-16]. Thus, NW is a considered a good sport for improving health.

One study [17] on adult women reported that oxygen uptake ( $\overset{\cdot }{V}O_{2}$) was approximately 20% higher during NW than during LW. Schiffer *et al*. [8] reported $\overset{\cdot }{V}O_{2}$ , heart rate (HR) and blood lactate concentration were significantly higher with NW than with LW at speeds above 1.8 m/s. In addition, their results showed that the differences in physiological response between NW and LW were smaller than in previous studies [7,11,17,18]. It has been consistently found that NW induces higher work intensity than LW, because of the propelling force produced by pushing the pole backward with the upper extremities [7,11,17,18]. However, the magnitude of physiological responses at different walking speeds during NW versus LW remains unclear, because of the wide variety of experimental conditions used in previous studies.

Willson *et al*. [16] indicated that NW induces a smaller inertia moment of the knee joint during the stance phase compared with LW, and suggested that this phenomenon may be related to the smaller load on the knees in NW. Schwameder *et al*. [19] indicated that the knee joint moment is reduced with the use of hiking poles during downhill walking. Based on these findings, NW is generally believed to reduce the load on the lower extremities by 20%. The findings of other studies [20,21] corroborate the findings of Willson *et al*. [16]. However, several recent studies [13-15] assessed motion analysis of subjects skilled in NW and reported that NW offered no biomechanical benefit for the lower extremities compared with LW. In fact, they [13-15] found that the load on the knee joint after a heel strike was actually higher in NW than in LW, and even suggested that NW should not be recommended for people who want to reduce biomechanical load during walking. However, in these studies on subjects skilled in NW, the experiments were conducted at relatively high walking speeds of 90 and 120 m/min, which are greater than the normal walking speeds used in daily life. Moreover, to determine whether NW alleviates the load on the lower extremities, it is important to compare integrated electromyography (iEMG) readings of the lower extremities between NW and LW at various speeds, including slower speeds (60 to 80 m/min). However, EMG studies during NW have been conducted at only one speed and inclination [2,22,23].

The conflicting results for NW obtained by previous studies could potentially be resolved through simultaneous measurement of $\overset{\cdot }{V}O_{2}$ and EMG activity via a speed-increment test during NW and LW. If NW has an alleviating effect on the lower extremities, then differences in iEMG between NW and LW would be detected in the lower extremities. This would also expand our basic knowledge about increases in $\overset{\cdot }{V}O_{2}$ during NW, especially if changes in iEMG readings of are detected.

The purpose of the present study was to measure the exercise intensity and EMG activity of the lower extremities in NW through a speed-increment test of adult subjects skilled in NW, and to clarify the physiological characteristics of NW.

## Methods

### Subjects

Ten healthy adults (four men, six women) were included in the present study. All subjects regularly engaged in physical exercise. All subjects were familiarized with the experimental procedures, and it was ensured that the attachment of the experimental device to the body did not have any effects on the physiological responses of the subjects during NW and LW. Prior to participation in this study, all subjects were fully informed of the study objectives, procedures, and possible risks of participation in the study, and all subjects gave written informed consent to participate. This study was approved by Human Ethics Review Committee of Juntendo University (approval No.24-66).

The subjects were asked to ensure that they received sufficient sleep and refrained from engaging in severe exercise and consuming alcohol 24 hours before the experiment. On the day of the experiment, each subject ingested a meal at least 2 hours before the walking tests, and arrived at the laboratory 1 hour before the test was scheduled to take place. To ensure that the subjects were in good physical condition, they underwent a medical examination, including completion of questionnaires and measurement of blood pressure, and their height, body weight, and percentage body fat were measured. The mean age, height, body weight, and percentage body fat were 21.8 ± 1.0 years, 1.75.8 ± 2.1 cm, 66.8 ± 4.4 kg and 14.4 ± 2.3%, respectively, in men, and 21.7 ± 1.5 years, 166.2 ± 4.0 cm, 61.0 ± 7.6 kg, and 25.3 ± 5.2%, respectively, in women (Table 1).

**Table 1:** Subject characteristics

**Subjects**	**Sex**	**Age, years**	**Stature, cm**	**Weight, kg**	**Body fat,%**
A	M	23	178.2	68.7	13.6
B	M	22	175.1	66.7	16.8
C	M	21	173.3	60.8	11.6
D	M	21	176.5	71.0	15.5
E	F	24	163.2	51.6	21.1
F	F	22	162.7	58.2	20.2
G	F	22	167.1	58.9	26.6
H	F	22	173.1	73.8	33.0
I	F	20	163.6	58.1	21.5
J	F	20	167.5	65.3	29.1
Mean ± SD	M	21.8 ± 1.0	175.8 ± 2.1	66.8 ± 4.4	14.4 ± 2.3
Mean ± SD	F	21.7 ± 1.5	166.2 ± 4.0	61.0 ± 7.6	25.3 ± 5.2

### Experimental conditions and length of Nordic pole

Each subject was taught the NW methods by an International Nordic Walking Federation (INWA)-qualified instructor (the master instructor) [1], and was familiarized with NW.

The pole length used for NW was selected and adjusted to permit a smooth arm motion, based on the INWA formula (0.68 × body height (in cm) [1], and to induce a near right-angle elbow flexion upon pole landing [19].

### Protocol

During the experiments, two submaximal walking tests consisting of NW and LW were performed by each subject. Thereafter, subjects were fitted with a respiratory mask for $\overset{\cdot }{V}O_{2}$ measurement, and with electrodes for electrocardiography (ECG) and electromyography (EMG) recordings. Resting $\overset{\cdot }{V}O_{2}$ and HR were determined over 5 minutes while the subject was sitting on a stool. After resting measurements were obtained, the subjects performed two submaximal walking tests, NW or LW, at random. Both walking tests were interspaced by a rest period, which permitted the physiological parameters and rating of perceived exertion, measured using the OMNI scale, to return to the resting values that were measured before the tests. Both tests began at an initial speed of 60 m/min for 3 minutes, followed by increases of 10 m/min every 2 minutes up to 120 m/min. During the tests, some physiological parameters were continuously measured over a 1-minute interval. Room temperature and relative humidity were 20.0 ± 1.8°C and 46.7 ± 12.9%, respectively.

### Oxygen uptake, ventilation, heart rate and OMNI scale

Throughout the tests, expired respiratory gas was analyzed using an automated metabolic analyzer (MG-360; Minato, Tokyo, Japan) [24], which had been calibrated with known concentrations of O_2_ and CO_2_ for fractional concentrations of both gases. In addition, expired gas volume was measured on a flow meter (RM-300; Minato) [25,26] every 30 seconds. $\overset{\cdot }{V}O_{2}$ and ${\overset{\cdot }{V}}^{E}$ were also calculated every 30 seconds during both tests. Perceived exertion was evaluated using the OMNI scale [27] during the final 15 seconds of NW and LW. As described by Robertson *et al*. [28], ratings of felt at the level of the whole body and the upper and lower extremities were evaluated, and all subjects were aware that these measurements were being evaluated to determine the stresses on the cardiorespiratory system and on the muscle groups of the upper and lower extremities, respectively. Perceived exertion was evaluated at rest and during each stage of the walking tests.

### Electromyogram recording and analysis

EMG activities of the upper and lower extremities were recorded from the thickest part of each muscle (the ‘belly’ of the muscle) of each muscle using pre-amplifier mounted electrodes (EMG Isolator SX230; Biometrics Ltd, Newport, UK). The skin over each muscle belly was wiped with pure ethanol, followed by skin preparation gel (Skin Pure; Nihon Kohden, Tokyo, Japan), to lower the resistance of the skin between the electrodes to less than 10 kΩ.

Based on the results of previous studies [23,29,30], EMG activities were recorded from four muscles in the left lower extremity and one muscle in the right upper extremity. Specifically, the muscles of lower extremity used for measurement were the vastus lateralis (VL), biceps femoris (BF), tibialis anterior (TA), and gastrocnemius (GA) of the left lower extremity, and the muscle of upper extremity was the triceps brachii (TB) of the right upper extremity.

**Figure 1:**
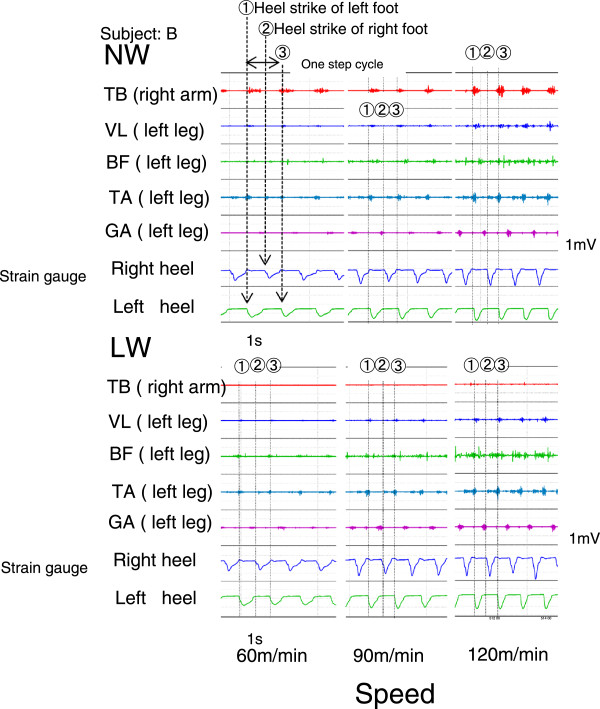
**Samples of electromyography (EMG) data and gait-cycle recording for a subject.** Upper panels show recording of Nordic walking (NW); lower panels show recording of level walking (LW). Left, middle and right panels indicate recordings at 60, 90 and 120 m/min, respectively. (1) and (3) show the heel strike of the left foot, and (2) shows the heel strike of the right foot. A gait cycle exists between (1) and (3), and the time between (1) and (2) includes the stance and push-off phases.

A one-step cycle during walking (gait-cycle) was measured by a strain-gauge board attached to the heels of both shoes. A gait-cycle was defined as the amount of time from a heel strike to the next heel strike on the same foot. A sample recording from a subject is provided in Figure 1.

Analog signals of the EMG instrument and the strain-gauge were sampled using an A/D converter (MP100; Biopac System Inc., Goleta, CA, USA) at 1 kHz, and were stored in a personal computer (iMac; Apple Corp, Cupertino, CA, USA). Digitized EMG signals were integrated over a 30-second period at each walking speed during NW and LW. Each iEMG signal was expressed as a percentage of the iEMG reading obtained during LW at 60 m/min for comparison.

### Statistical analysis

All data are expressed as mean ± SD. $\overset{\cdot }{V}O_{2}$, ${\overset{\cdot }{V}}^{E}$, HR and iEMG data obtained during the final 30 seconds at each walking speed were used to compare differences between the two walking methods (NW and LW). All statistical analyses were conducted using Statview software (version 5.0; SAS Institute Inc., Cary, NY, USA). Two-way analysis of variance (ANOVA) analyzing walking speeds and walking with or without Nordic poles, was used to determine the significant differences between walking methods at different speeds. If a significant *F* ratio was indicated, Fisher’s protected least squares difference was used as a *post hoc* test to determine the significance of differences. *P* < 0.05 was considered significant.

## Results

### Oxygen uptake, ventilation, heart rate, and OMNI scale

**Figure 2:**
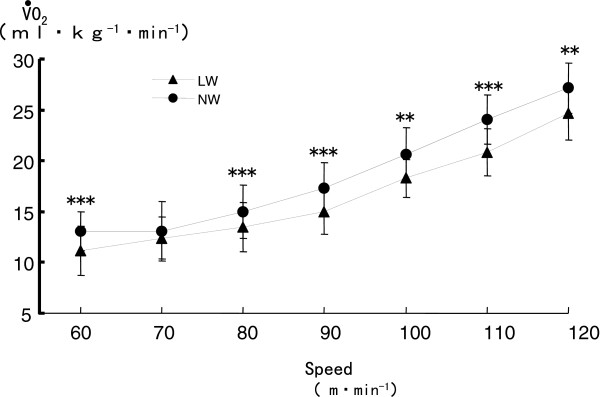
**Comparison of oxygen uptake (**$\overset{\cdot }{V}O_{2}$**) during Nordic walking (NW) and level walking (LW).** **P < 0.01, *** *P* < 0.001.

**Figure 3:**
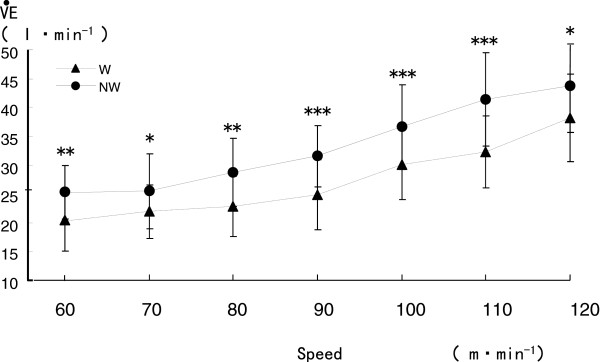
**Comparison of ventilation (**${\overset{\cdot }{V}}^{E}$**) during Nordic walking (NW) and level walking (LW).** *P < 0.05, **P < 0.01, ***P < 0.001.

**Figure 4:**
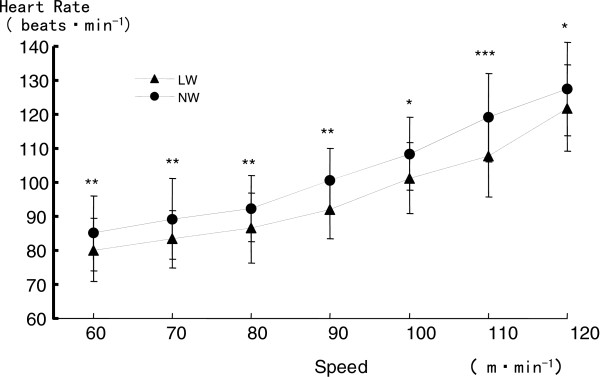
**Comparison of heart rate (HR) between Nordic walking (NW) and level walking (LW).** *P < 0.05, **P < 0.01, ***P < 0.001.

**Figure 5:**
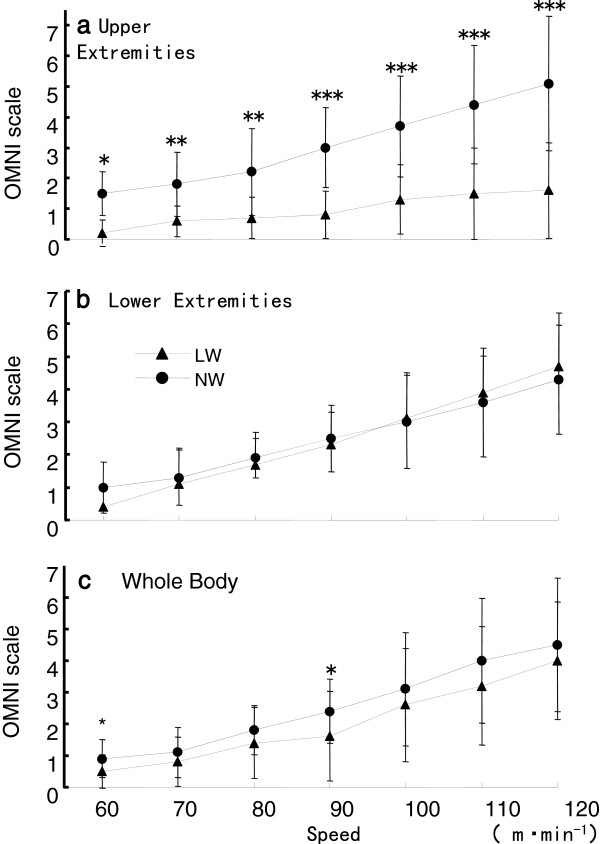
**Comparison of perceived exertion (OMNI scale) for Nordic walking (NW) and level walking (LW).(A)** Upper extremities, **(B)** Lower extremities, **(C)** Whole body. *P < 0.05, **P < 0.01, ***P < 0.001.

$\overset{\cdot }{V}O_{2}$, ${\overset{\cdot }{V}}^{E}$, HR and OMNI scale values at each walking speed during NW and LW were assessed. $\overset{\cdot }{V}O_{2}$ during NW was significantly greater than that during LW, except at a speed of 70 m/min (*P* < 0.01) (Figure 2). ${\overset{\cdot }{V}}^{\overset{\cdot }{E_{V}}}$ during NW was significantly greater than that for LW in 3.6 to 9.1 l/min at all speeds (*P* < 0.05 to 0.001) (Figure 3). Mean HR during NW was 2–7 beats/min greater than that during LW (*P* < 0.05 to 0.001) (Figure 4). The OMNI scale for the whole body was increased from 0.5 ± 0.5 at 60 m/min to 4.0 ± 1.9 at 120 m/min during LW, and from 0.9 ± 0.6 at 60 m/min to 4.5 ± 2.1 at 120 m/min during NW (*P* < 0.05) (Figure 5). The OMNI scale for the lower extremities was not different between NW and LW at the same walking, however, for the upper extremities, the OMNI scale for the lower extremities was similar at all walking speeds for LW, but increased during NW, from 1.5 ± 0.7 at 60 m/min to 5.1 ± 2.1 at 120 m/min. The differences in OMNI scale between NW and LW for the upper extremities at each walking speed were significant (*P* < 0.05).

### Integrated electromyography showing percentage change from level walking

**Figure 6:**
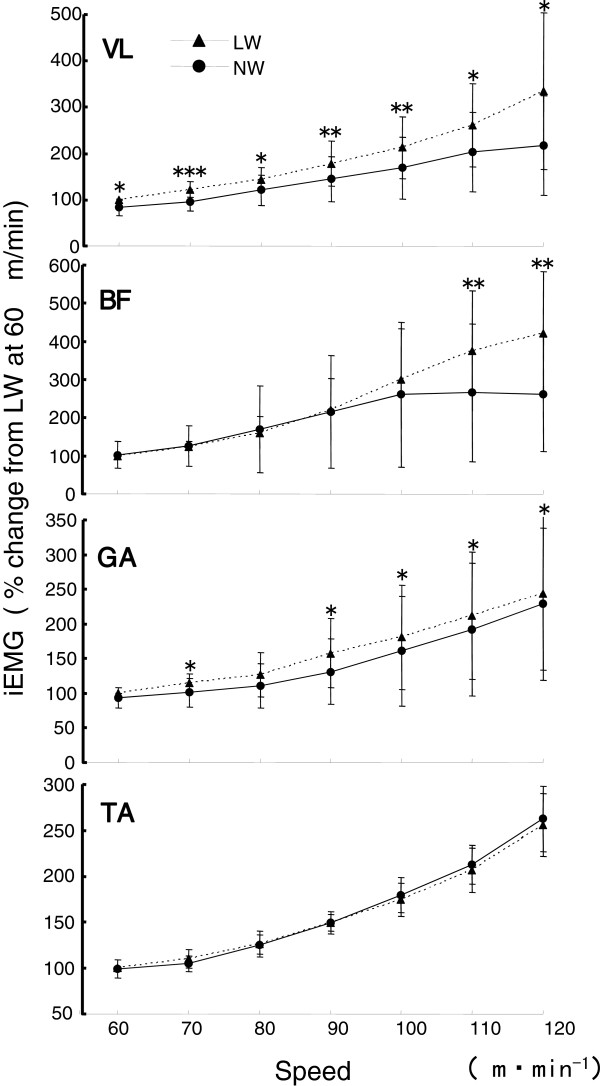
**Comparison of the integrated electromyography (iEMG) readings from leg muscles during exercise.** The iEMG of four muscles in the lower extremity (vastus lateralis (VL), biceps femoris (BF) and gastrocnemius (GA) and tibialis anterior (TA)) during Nordic walking (NW) and level walking (LW). *P < 0.05, **P < 0.01, ***P < 0.001.

**Figure 7:**
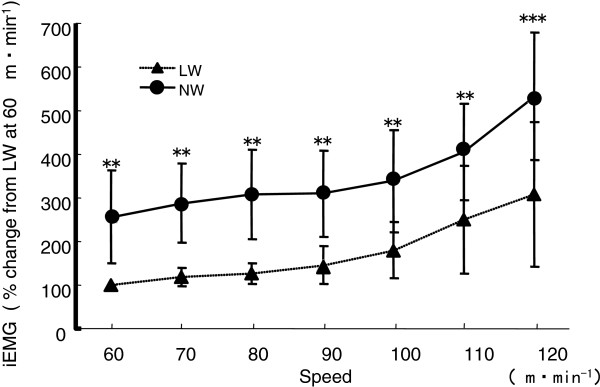
**Comparison of the integrated electromyography (iEMG) of readings from the triceps brachii was measured during each type of walking.** **P < 0.01, ***P < 0.001.

The iEMG readings recorded for the VL, BF, GA, TA and TB muscles showed differences between NW and LW (Figure 6). The iEMG of VL was lower during NW than during LW at all walking speeds (*P* < 0.05), and the difference between NW and LW became greater as the walking speed increased (−83.7 ± 18.6% during NW versus 100% during LW at 60 m/min, and 217.2 ± 107.8% during NW versus 334.8 ± 168.7% during LW at 120 m/min). The iEMG of the BF was significantly lower during NW than during LW at 110 and 120 m/min (*P* < 0.01), and significantly lower iEMG values were found for the GA during NW versus LW at walking speeds of 70, 90, 100 and 120 m/min (*P* < 0.05). The iEMG values of the TA increased with increasing walking speeds for both NW and LW, there were no significant differences the two types of exercise. Conversely, the iEMG of the TB during both walking conditions were broadly similar for speeds of 60 to 90 m/min, and increased at walking speeds of greater 100 m/min. Differences in iEMG between NW and LW were significant at all walking speeds (*P* < 0.01) (Figure 7).

## Discussion

The primary purpose of the present study was to clarify the physiological characteristics of NW at speeds between 60 to 120 m/min on a treadmill. The iEMG readings for the revealed an interesting phenomenon; at all walking speeds, the iEMG of VL during NW was significantly lower than that during LW, and the difference increased markedly with increasing speed. Moreover, the iEMG of the BF muscle during NW was also significantly lower than that during LW at 110 and 120 m/min.

These results corroborate the findings of Knight and Caldwell [22], who reported a marked discharge in the VL from the early to middle stance phase, whereas there was a discharge in the BF from the late swing phase to the next early stance phase, under inclining conditions. In the present study, NW induced a significant reduction in the iEMG of the GA at all walking speeds compared with LW. The recording data on EMG for each subject showed that the TB was activated and that the muscles of lower extremities (VL, BF and GA) tended to show lower activity compared with LW in the stance and push-off phases during NW (Figure 1). These data support the notion that the pulling-off activity in the push-off phase of NW reduces the load on the lower extremities during the stance phase compared with the load during LW [2,16]. These findings also must be related to the finding that the pulling-off activity in the push-off phase attenuates oxygen uptake in the lower extremities during NW. In fact, EMG activity of the TB reportedly increases during the stance phase, and reaches a peak value in the push-off phase during NW [23]. In addition, iEMG findings with respect to the TB in the present study were significantly higher during NW than during LW. Furthermore, the decreased iEMG for the VL and GA muscles found at 60 m/min suggests that NW is an effective exercise for people with atrophy of the lower extremities. These findings partly support the observations of a previous study in which the load on the lower extremities of patients with intermittent claudication was decreased at a slower speed of 53 m/min [21]. Hence, it is suggested that using the poles during NW not only increases energy expenditure, but also reduces the load on the during the stance and push-off phases.

By contrast, despite the reductions in the iEMG readings for the lower extremities, ${\overset{\cdot }{V}}^{E}$ and HR were found to be higher during NW than during LW at all given speeds, and $\overset{\cdot }{V}O_{2}$ was also found to be significantly higher during NW than during LW at all speeds except 70 m/min. $\overset{\cdot }{V}O_{2}$ during NW increased by 12% to 19% at all speeds, in addition to the significant increases in HR. These findings are in line with the observations of previous reports. NW thus seems to increase energy expenditure by using the upper body during exercise, even at lower speeds of 60 m/min. This is similar to findings of previous studies [7,10,11,17], which found that the physiological demands during NW clearly differ from those during LW. For example, Church *et al*. [17] reported that NW induced significantly higher responses for HR (6%) and $\overset{\cdot }{V}O_{2}$ (21%) during LW at 93 m/min on a 200 meter track. Rodger *et al*. [7] reported that $\overset{\cdot }{V}O_{2}$ was 12% higher during NW than during LW at 112 m/min on a treadmill. Furthermore, Tomita *et al*. [10] and Walter *et al*. [11] found that NW on level ground induced $\overset{\cdot }{V}O_{2}$ responses that were 9% and 21% higher, $\overset{\cdot }{V}O_{2}$ respectively, compared with LW.

To our knowledge, only one previous study has presented findings that conflict with those of the present study. Schiffer *et al*. [8] found no differences in $\overset{\cdot }{V}O_{2}$ and HR at speeds of less than 98 m/min. This discrepancy may exist because that study used a protocol in which the speed was increased rapidly from 70 to 126 m/min within 5 minutes, by increments of 18 m/min every minute on level ground. Therefore, the physiological parameters at speeds of less than 100 m/min would not necessarily produce steady-state values because the incremental speed increases were made too rapidly. Subjects in the present study walked at 60 m/min for 3 minutes, and performed a ramp exercise up to 120 m/min with increases in speed of 10 m/min every 2 minutes. In other words, our outcomes reflect the physiological responses during NW at each speed.

The OMNI scale of the whole body, which rates perceived exertion, was significantly higher during NW than during LW only at speeds of 60 and 90 m/min, and these differences were much smaller than those for $\overset{\cdot }{V}O_{2}$ and HR. These results resemble the findings of previous studies [10,17], in that the physiological responses were higher during NW than during LW, even when ratings did not increase. However, the OMNI scale for the upper extremities was found to be significantly higher during NW than during LW at all speeds. Considering the iEMG of the TB, this muscle group in the upper extremities would increase the perceived exertion, whereas the OMNI scales for the lower extremities and the whole body would not differ significantly. Additionally, $\overset{\cdot }{V}O_{2}$ and iEMG activity of the TB during NW were significantly increased at all speeds compared with those during LW, whereas iEMG activities of BF and VL were not increased at speeds >90 m/min. These phenomena illustrate that the rate of energy expenditure for the may be greater than the differences in $\overset{\cdot }{V}O_{2}$ between NW and LW.

**Figure 8:**
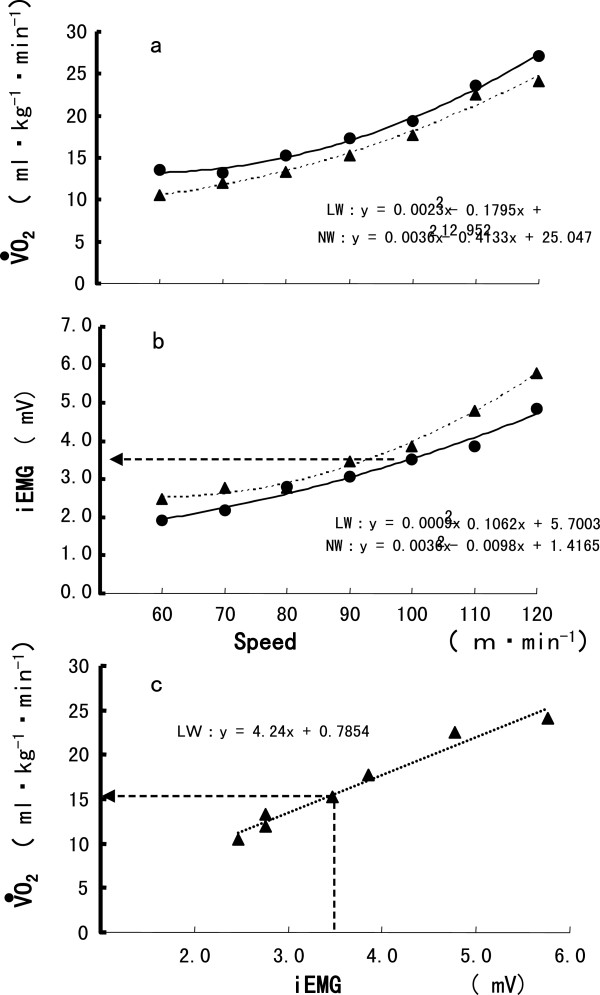
**Estimation of the relationship between various parameters.** The relationship between **(A)** oxygen uptake ( $\overset{\cdot }{V}O_{2}$) and speed, **(B)** total integrated electromyography (iEMG) of lower extremity muscles and speed during Nordic walking (NW) and level walking (LW), and **(C)**$\overset{\cdot }{V}O_{2}$ and total iEMG of leg muscles during LW of the subject. The $\overset{\cdot }{V}O_{2}$ of the lower extremity muscles during NW can be estimated from the numerical linear formulas (C) for $\overset{\cdot }{V}O_{2}$ and iEMG during LW.

Naturally, differences in $\overset{\cdot }{V}O_{2}$ of the lower extremities would be decreased, and the distribution of energy expenditure for each part of the body needs to be clarified. The correlation between the amount of iEMG discharge and the rate of oxygenated hemoglobin is negative [31]. Additionally, Bigland-Ritchie *et al*. [32] showed a strong linear relationship between $\overset{\cdot }{V}O_{2}$ and iEMG. Moreover, Bojsen *et al*. [33] used positron emission tomography to evaluate muscle-activity patterns during double poling on a ski ergometer, and they found that the muscles of upper extremities, abdominal muscles, and hip flexors had the highest glucose uptake index during poling. NW shows similarities to cross-country skiing in terms of use. Therefore, it should be possible to estimate the distribution of energy expenditure for each part of the body from the relationship between $\overset{\cdot }{V}O_{2}$ and iEMG in this study. We estimated the rate of $\overset{\cdot }{V}O_{2}$ of the lower extremities during NW by using the iEMG during NW at each speed in the $\overset{\cdot }{V}O_{2}$ -iEMG relationship formula for LW, and calculating $\overset{\cdot }{V}O_{2}$ of the (example shown in Figure 8). From this, we estimate that 20 to 30%; of the total $\overset{\cdot }{V}O_{2}$ accounted for the energy expenditure without the volume of the lower extremities. Considering the significantly increased ${\overset{\cdot }{V}}^{E}$ and iEMG of TB during NW, 20 to 30%; of the total $\overset{\cdot }{V}O_{2}$ would be used by the muscles of the upper body and respiratory system during NW.

Interestingly, no significant differences in $\overset{\cdot }{V}O_{2}$ were seen during NW and LW at 70 m/min, and $\overset{\cdot }{V}O_{2}$ during NW showed no linear increases between 60 and 120 m/min. $\overset{\cdot }{V}O_{2}$ and iEMG during NW produced a U-shaped curve against speed, which is similar to the relationship between $\overset{\cdot }{V}O_{2}$ and speed during walking [34]. It is also interesting to note that a small difference existed in $\overset{\cdot }{V}O_{2}$ between NW and LW at 70 m/min. We illustrated the relationship between $\overset{\cdot }{V}O_{2}$ and total iEMG of the leg muscles at all speeds for subjects (example shown in Figure 8A, B). Changes in $\overset{\cdot }{V}O_{2}$ induce changes in iEMG during NW, and this relationship was clearly demonstrated in a previous study [34]. This relationship was seen in almost all subjects, suggesting that each individual has an optimally economical speed for NW. Thus, the optimal speed during NW should be considered for each walker.

## Conclusion

NW was found to induce higher $\overset{\cdot }{V}O_{2}$, HR and iEMG of the upper extremities at all walking speeds compared with LW. ${\overset{\cdot }{V}}^{E}$ during NW was significantly greater than that for LW at all speeds. The iEMG readings for the lower extremities were significantly lower during NW than during LW. These data indicate that the use of poles in NW attenuates the muscle activity of the lower extremities during the stance and push-off phases, and decreases energy expenditure of the lower extremities and increase energy expenditure of the upper body and respiratory system at certain walking speeds.

## Abbreviations

$\overset{\cdot }{V}O_{2}$: Oxygen uptake; HR: Heart rate; EMG: Electromyogram; iEMG: Integrated electromyography; NW: Nordic walking; LW: Level walking; TB: Triceps brachii; VL: Vastus lateralis; BF: Biceps femoris; GA: Gastrocnemius; TA: Tibialis anterior.

## Competing interests

The authors declare that they have no competing interests.

## Authors’ contributions

KS and MK designed and coordinated the study, carried out the experiment, and drafted the manuscript. HT helped to analyze the data and participated in the design of study. SK coordinated and helped to draft the manuscript. All authors read and approved the final manuscript.
